# Diabetes Mellitus Secondary to Acute Pancreatitis in a Child with Wolf-Hirschhorn Syndrome

**DOI:** 10.1155/2017/3892467

**Published:** 2017-10-24

**Authors:** Asma Deeb

**Affiliations:** Paediatric Endocrinology Department, Mafraq Hospital, P.O. Box 2951, Abu Dhabi, UAE

## Abstract

Wolf-Hirschhorn Syndrome (WHS) is a rare genetic disease caused by deletion in the short arm of chromosome 4. It is characterized by typical fascial features and a varying degree of intellectual disabilities and multiple systemic involvement. Epidemiological studies confirmed the association of acute pancreatitis with the development of diabetes. However, this association has not been reported in WHS. We report an 18-year-old girl with WHS who presented acutely with nonketotic Hyperglycemic Hyperosmolar Status (HHS) in association with severe acute pancreatitis. Her presentation was preceded by febrile illness with preauricular abscess. She was treated with fluids and insulin infusion and remained on insulin 18 months after presentation. Her parents are cousins and the mother was diagnosed with type 2 diabetes. She had negative autoantibodies and no signs of insulin resistance and her monogenic diabetes genetic testing was negative. Microarray study using WHS probe confirmed deletion of 4p chromosome. Acute pancreatitis is uncommon in children and development of diabetes following pancreatitis has not been reported in WHS. HHS is considerably less frequent than diabetes ketoacidosis in children. We highlight the complex presentation with HHS and acute pancreatitis leading to diabetes that required long term of insulin treatment.

## 1. Introduction

Cooper and Hirschhorn first documented Wolf-Hirschhorn syndrome (WHS) in 1961. The syndrome is caused by a molecular deletion in the short arm of chromosome 4 (4p). It is characterized by the typical fascial features of the Greek warrior helmet appearance of the nose and forehead. Patients with WHS have a varying degree of intellectual disabilities and systemic involvement [1].

The American Diabetes Association classified diabetes resulting from exocrine damage as type 3c. The exocrine damage can be related to pancreatitis, cystic fibrosis, hemochromatosis, pancreatic cancer, pancreatectomy, and pancreatic agenesis [2].

A meta-analysis highlighted the risk of diabetes following pancreatitis. 24 prospective studies of 1,102 patients with pancreatitis were studied. 37% developed prediabetes or diabetes. 16% who developed diabetes needed insulin. It was shown that a diagnosis of acute pancreatitis increases the risk of developing diabetes by over twofold over 5 years [3]. Pooled prevalence of newly diagnosed diabetes within 1 year was 15% and increased to 40% after 5 years of the acute pancreatitis.

A population-based study examined the risk of diabetes after pancreatitis. The study included 2996 patients. Incidence of diabetes was 60.8 per 1000 indicating a twofold increase of developing diabetes [4]. The study showed that the risk of developing diabetes is higher in young males of under 45 years.

To the best of our knowledge, this is the first report of a child with WHS who developed diabetes following acute pancreatitis.

## 2. Clinical Presentation

The patient is an 18-year-old girl who presented acutely with severe abdominal pain, diarrhea, and vomiting. She was dehydrated and had acidotic breathing. She had hyperglycemia (glucose of 40 mmol/720 mg) and hypernatremia (sodium of 176 mmol). Inflammatory markers were high: procalcitonin 7.550 ng/ml and CRP of 179. Her white cell count was 24.2 × 10^9^/l (NR 4–11) with 65.3% neutrophilia and 8.4% monocytosis. She had severe acidosis (pH of 6.95) with no ketosis. Her serum amylase was 354 IU/L (NR 28–100) and serum lipase was 5739 IU/L (NR 13–60). LDH was high at 502 (NR 135–225). Her urea was high at 20.9 mmol/L (NR < 8.3), creatinine 269 *μ*mol/L (NR 45–84), and GFR 15 ml/min/1.73 m^2^. Liver function tests were normal as was the triglyceride level. Blood, stool, and urine cultures were negative. CT abdomen excluded intra-abdominal abscesses. Chest X-ray showed blunting of both costophrenic angles with no other pulmonary features suggestive of inflammation or pneumonia. Chest CT confirmed bilateral pleural effusion. The diagnosis was reached as severe acute pancreatitis complicated by HHS.

### 2.1. Past Medical History

**Figure 1:**
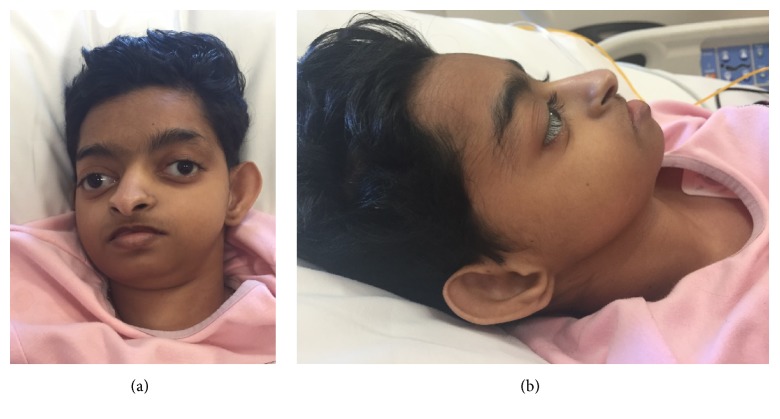
Front and side fascial photo of the patient showing characteristic features of WHS (Roman helmet appearance, microcephaly, proptosis, short philtrum, high-arched eyebrows, and big ears with minimal creases).

**Figure 2:**
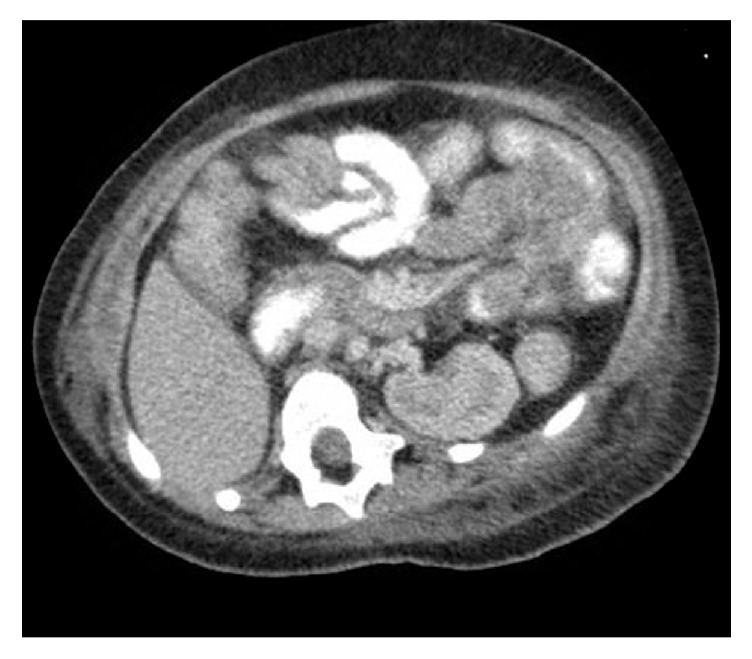
Abdominal CT scan showing solitary left kidney with kidney cyst and nephrocalcinosis.

The patient was diagnosed clinically with WHS. She has the typical fascial features of the syndrome (Figures 1(a) and 1(b)) and had severe learning disability and global developmental delay. She has microcephaly with marked brain atrophy and ventricular dilatation and suffered from epilepsy. Her seizures were well-controlled on valproic acid and levetiracetam. She has a solitary ectopic kidney with nephrocalcinosis and chronic renal failure (Figure 2). Six weeks prior to the acute presentation, she was febrile with a preauricular abscess. The abscess was drained and she was started on antibiotics. Inflammatory markers were high but her abscess fluid showed no bacterial growth. She remained unwell for about 6 weeks until she presented with the acute illness.

### 2.2. Family History

Parents are second-degree cousins of Yemeni origin. The mother, who is 52 years, was diagnosed with type 2 diabetes at the age of 45 and is on multiple oral hypoglycemic agents. Her BMI is 28 kg/m^2^ and her HbA1c is 9%. She has 4 healthy siblings.

### 2.3. Treatment

On the acute presentation, she was treated for the HHS. She was rehydrated with normal saline and put on insulin infusion initially at 0.05 unit/kg/day. Her clinical status improved, glucose normalized, and she was shifted to subcutaneous insulin as basal bolus regime with an insulin requirement of 0.3 unit/kg. She was treated conservatively for acute pancreatitis and showed progressive clinical improvement. Her insulin requirement continued to drop and she started having hypoglycaemia. Insulin was stopped and she was discharged home off insulin.

### 2.4. Progress

Three weeks after discharge, she presented acutely with HHS. She was readmitted and restarted on insulin. After recovery, she was discharged home on insulin glargine and insulin aspart. Her fasting glucose ranged between 90 and 127 mg and postprandial glucose between 110 and 138 mg. She experienced no hypoglycaemia and remained on 0.3 units/kg/day of insulin.

## 3. Further Investigations

### 3.1. Biochemistry

Initial HbA1c was 8.4%. Following treatment, it went down to 6.9% in 3 months and to 5.4% in 6 months. Her latest HbA1c (18 months after the initial presentation) is 6.5%. Her initial C-peptide was 0.11 nmol (NR 0.37–1.47). After 6 months, it was 0.3 nmol. Her autoantibodies to GAD, IA2, and insulin were negative.

### 3.2. Microarray Analysis

Microarray analysis was performed using the critical region probe (WHSCR). FISH analysis on the metaphase spreads and the interphase nuclei showed abnormal signal pattern for the WHSCR probe with deletion in all cells examined (100 cells). These results are diagnostic for WHS.

### 3.3. Genetic Test for Monogenic Diabetes

Analysis of all coding regions and exon/intron boundaries of the* HNF1β* gene by Sagner sequencing was done. Dosage analysis of* GCK, HNF4A, and HNF1B* by MLPA using MRC-Holland kit P241-D1 was performed. Analyses did not identify a pathogenic mutation or partial/whole gene deletion.

## 4. Discussion

The patient presentation was complex. In addition to her underlying WHS and chronic renal failure, she has a picture of severe acute pancreatitis. The underlying cause of the pancreatitis was not established. However, her preceding presentation with preauricular sterile abscess suggested viral parotitis with possible pancreatic involvement. Her extreme elevation in glucose and hyperosmolality without ketosis are characteristic of HHS. HHS is considerably less frequent in children than diabetes ketoacidosis [5]. Unlike HHS in adults, where comorbidity conditions are seen, paediatric HHS occurs most often in otherwise healthy children and adolescents with type 2 diabetes particularly obese males [5]. These known facts about HHS added complexity to her presentation and diagnosis.

On the initial presentation, her hyperglycemia was thought to be “stress hyperglycemia” due to the severity of pancreatitis. However, glucose was very high and associated with acidosis requiring insulin infusion. As her HbA1c was high and she remained requiring insulin, it became apparent that she had diabetes rather than transient stress hyperglycemia.

High glucose has been integrated in the scoring to assess the severity of acute pancreatitis. Elevated pancreatic enzymes can be seen in decompensated diabetes; however, only 11% of cases with diabetes ketoacidosis display elevated enzymes and the radiological features of pancreatitis in imaging [6].

The ideal fluid choice in acute pancreatitis management is debatable. Our patient was resuscitated with normal saline fluid boluses. Some references suggest using Ringer lactate solution might be superior in acute pancreatitis [7]. The rationale for using Ringer lactate is its anti-inflammatory effect that can be beneficial in pancreatitis.

The link between acute pancreatitis and development of diabetes is debatable. Studies showed that patients who had surgery for acute pancreatitis have a 28–100% chance of developing diabetes [8] while the risk is lower with conservative treatment (6.2–15.7%) [9]. These observations suggested that acquiring diabetes is secondary to surgical intervention. However, Das et al. showed that neither the treatment modality nor the severity is detrimental in developing diabetes [3]. Over 30% of patient who had an episode of pancreatitis develop glucose intolerance, impaired *β*-cell function, and insulin resistance even after mild pancreatitis [10].

Developing diabetes concurrently or shortly after severe pancreatitis can be explained by the extensive necrosis of the pancreas with severe acute pancreatitis which is the case in our patient. However, developing diabetes shortly after mild pancreatitis might be less plausible.

Das et al. proposed that pancreatitis might not be causative but a triggering insult with certain predisposing factors including autoimmunity, genetic susceptibility, or certain metabolic factors such as obesity and dyslipidemia. Our patient had a negative autoimmune screen; her BMI was 16.33 kg/m^2^ (−2.66 SDS) with no clinical features of insulin resistance and normal lipid profile. These, collectively, make the diagnosis of type 1 and type 2 diabetes unlikely. Due to lack of autoimmune markers and the history of consanguinity, monogenic diabetes was considered. As she has a kidney cyst, the possibility of MODY 5 (renal cyst and diabetes syndrome) was raised. However, testing for genetic causes of monogenic diabetes, including HNF1*β*, was negative. Accordingly, we postulate that the pancreatitis was causal of the diabetes. On presentation, her HbA1c was 8.4% indicating that her glucose intolerance was existing prior to her presentation. She was unwell for few weeks prior to presentation and we presume that she could have had subacute pancreatitis which was not picked up considering the severity of the child disability and lack of communication.

An additional thought about the link of diabetes and WHS is related to the C4orf48 which is a gene located in the locus of WHS. C4orf48 is important for cell differentiation. High level of this gene is expressed in the pancreatic tissue postnatally [11]. It is intriguing whether deletion of the WHS locus might be related to dysfunction of pancreatic cell differentiation and causation (or predisposition) of diabetes.

## Additional Points

(i) This is the first case report of a patient with WHS who developed diabetes mellitus following acute pancreatitis. (ii) Diabetes can present as HHS in adolescents following severe pancreatitis. (iii) Establishing the cause of diabetes is important for proper treatment and follow-up.
